# Editorial: Nurturing the brain: Associations between family environment and child brain development

**DOI:** 10.3389/fnins.2023.1119838

**Published:** 2023-01-31

**Authors:** Sandra Thijssen, Madelon M. E. Riem, Nandita Vijayakumar, Maaike J. Cima, Sarah Whittle

**Affiliations:** ^1^Behavioral Science Institute, Radboud University, Nijmegen, Netherlands; ^2^School of Psychology, Deakin University, Melbourne, VIC, Australia; ^3^VIGO, Child and Youth Care Institute, Nijmegen, Netherlands; ^4^Melbourne Neuropsychiatry Centre (MNC), Department of Psychiatry, The University of Melbourne & Melbourne Health, Melbourne, VIC, Australia

**Keywords:** family, parenting, brain development, socio-economic status, MRI, fMRI, EEG

Parental care plays a vital role in a child's neural and behavioral development. However, most research on the association between family factors and neurodevelopment focuses on extreme adversities such as child maltreatment (e.g., Riem et al., 2015; Rakesh et al., 2021). These studies may not generalize to the larger population of children growing up in more normative environments. Moreover, the majority of research on family emphasizes proximal (e.g., parenting) or distal factors [e.g., socio-economic status (SES)] whereas intermediate factors (e.g., family dynamics) or interactions between family factors may also explain variation in neurodevelopment.

Inspired by Bronfenbrenner's bio-ecological model (Bronfenbrenner, 1977)—which categorizes aspects of the child's environment according to the proximity to the child, and suggests environments interact to influence child development—this Research Topic sought to answer the following questions: (1) How do different levels of the family environment, ranging from proximal to distal factors, affect child neurodevelopment and consequently behavioral development? (2) Does their effect depend on the developmental period in which they occur or on the period in which the effects are measured?

Several studies in this Research Topic examined the association between parenting—a proximal factor of the family environment—and child neurodevelopment. Copeland et al. examined whether maternal sensitivity at 8 months was associated with resting-state functional connectivity (FC) at 8 (*n* = 17) and 30 (*n* = 39) months. Findings suggest that maternal sensitivity is associated with regional homogeneity (i.e., local FC with neighboring nodes) in the medial prefrontal cortex at 8, but not 30, months.

Similarly, Richmond et al. examined parenting and structural rather than functional network properties. They specifically investigated the relationship between positive and negative parenting behaviors and the development of structural networks over an 18-month period in 114 children aged 8–10. No associations were found between parenting and network development. However, less positive, but not more negative, parenting was associated with higher modularity (i.e., higher network segregation) at age 10. As modularity increases from childhood throughout adolescence (Khundrakpam et al., 2017), these results support prior research suggesting that a lack of positive parenting relates to accelerated neurodevelopment (Thijssen et al., 2017).

Extending beyond parent-child interactions, Coughlin et al. examined dynamics in the broader family and related this to hippocampal structure in 7–12 y.o. children (*n* = 91) and adults (*n* = 58). A positive perception of family interactions was associated with greater CA1 and CA2/3 hippocampal volumes, subfields previously shown to be sensitive to social stimuli (Lin et al., 2018; Cilz et al., 2019). Interestingly, family dynamics predicted hippocampal subfields across child *and* adult participants, suggesting that the hippocampus tracks fluctuations in family dynamics across the lifespan. Comparatively, the distal family factor SES was not associated with hippocampal structure.

Like Coughlin et al., Tyborowska et al. examined hippocampal subfield volume. Using longitudinal data from a community sample of mother–child dyads (*N* = 73), they examined the role of maternal prenatal (third trimester) cortisol concentrations on subsequent gray matter volume in 12 year-olds. Null findings emerged across markers of cortisol levels and several brain structures (whole brain, amygdala and hippocampus, hippocampal subfield volumes). Accordingly, variations in maternal late pregnancy cortisol concentrations may not be related to brain structure at puberty onset. Alternatively, effects may be small, or other aspects of the early life environment may disguise effects of prenatal stress.

Chajes et al. examined the interplay between macro- and micro-environments of the child. They investigated the relationship between SES and FC in 5-month old infants, and explored whether maternal sensitivity mediates or moderates that relationship. No direct links between SES and FC were found, nor did sensitive caregiving mediate or moderate the effects of SES. The authors propose SES-related effects may emerge only later in development and cannot yet be detected at 5 months post-partum. Interestingly, maternal sensitivity was significantly related to FC within the default mode network. Similar to Copeland et al.'s findings of associations between sensitivity and FC at 8 months, these results suggest infancy may be a sensitive period for maternal sensitive behavior and indicate a possible early-emerging neural mechanism underlying the link between early caregiving experiences and later social-emotional functioning.

Finally, Mulligan et al. examined how the family environment and child neural functioning interact to predict child behavior in a low SES urban sample. Specifically, they examined whether the relationship between daily parenting hassles and externalizing behaviors depends on the child's frontal alpha asymmetry during a frustration task. Findings suggest that daily hassles only relate to externalizing behaviors in children with high left frontal asymmetry. This effect was consistent with differential susceptibility: in children with high asymmetry, low parenting hassles were protective against externalizing behaviors, whereas high levels of parenting hassles formed a risk-factor for externalizing behavior.

This Research Topic covered a wide variety of familial factors and neural outcomes. With regards to the first research question, the studies suggest stronger associations between proximal factors of family (e.g., parenting) and brain development, than distal factors (e.g., SES) (Chajes et al.; Coughlin et al.). Moreover, based on different results for positive and negative parenting behaviors, findings suggest that the absence of positive parenting is functionally different from the presence of negative parenting (Richmond et al.). Not only may different factors have different effects, but in answering research question two, this Research Topic also provides evidence that the same family factor may have different effects across development (Copeland et al.; Richmond et al.), suggesting the existence of sensitive periods (Copeland et al.; Chajes et al.) as well as long-term effects (Richmond et al.). Finally, brain functioning may not only mediate associations between the family environment and child behavior, but may also affect the way in which the family environment affects child development (Mulligan et al.). Most importantly, the variety of results presented in this Research Topic illustrates the relevance of family neurobiology research and inspires new questions regarding the interactions between family factors and their timing. Finally, future consideration of animal work may help us to better understand mechanisms.

## Author contributions

ST, MR, NV, and SW drafted the manuscript. All authors provided feedback and helped revise the manuscript. All authors contributed to the article and approved the submitted version.
